# Integration of ARTP Mutation and Adaptive Laboratory Evolution to Reveal 1,4-Butanediol Degradation in Pseudomonas putida KT2440

**DOI:** 10.1128/spectrum.04988-22

**Published:** 2023-04-17

**Authors:** Xiujuan Qian, Kaiyuan Xin, Lili Zhang, Jie Zhou, Anming Xu, Weiliang Dong, Min Jiang

**Affiliations:** a Key Laboratory for Waste Plastics Biocatalytic Degradation and Recycling, College of Biotechnology and Pharmaceutical Engineering, Nanjing Tech University, Nanjing, People’s Republic of China; b State Key Laboratory of Materials-Oriented Chemical Engineering, Nanjing Tech University, Nanjing, People’s Republic of China; State Key Laboratory of Microbial Resources, Institute of Microbiology, Chinese Academy of Sciences

**Keywords:** 1,4-butanediol, *Pseudomonas putida* NB10, ARTP, ALE, metabolic mechanism, GCL pathway

## Abstract

Biotransformation of plastics or their depolymerization monomers as raw materials would offer a better end-of-life solutions to the plastic waste dilemma. 1,4-butanediol (BDO) is one of the major depolymerization monomers of many plastics polymers. BDO valorization presents great significance for waste plastic up-recycling and fermenting feedstock exploitation. In the present study, atmospheric pressure room temperature plasma (ARTP)-induced mutation combined with adaptive laboratory evolution (ALE) was used to improve the BDO utilization capability of Pseudomonas putida KT2440. The excellent mutant P. putida NB10 was isolated and stored in the China Typical Culture Preservation Center (CCTCC) with the deposit number M 2021482. Whole-genome resequencing and transcriptome analysis revealed that the BDO degradation process consists of β-oxidation, glyoxylate carboligase (GCL) pathway, glyoxylate cycle and gluconeogenesis pathway. The imbalance between the two key intermediates (acetyl-CoA and glycolyl-CoA) and the accumulation of cytotoxic aldehydes resulted in the weak metabolism performance of KT2440 in the utilization of BDO. The balance of the carbon flux and enhanced tolerance to cytotoxic intermediates endow NB10 with great BDO degradation capability. This study deeply revealed the metabolic mechanism behind BDO degradation and provided an excellent chassis cell for BDO further up-cycling to high-value chemicals.

**IMPORTANCE** Plastic waste represents not only a global pollution problem but also a carbon-rich, low-cost, globally renewable feedstock for industrial biotechnology. BDO is the basic material for polybutylene terephthalate (PBT), poly butylene adipate-co-terephthalate (PBAT), poly (butylene succinate) (PBS), etc. Herein, the construction of BDO valorization cell factory presents great significance for waste plastic up-recycling and novel fermentation feedstock exploitation. However, BDO is hard to be metabolized and its metabolic pathway is unclear. This study presents a P. putida mutant NB10, obtained through the integration of ARTP and ALE, displaying significant growth improvement with BDO as the sole carbon source. Further genome resequencing, transcriptome analysis and genetic engineering deeply revealed the metabolic mechanism behind BDO degradation in P. putida, this study offers an excellent microbial chassis and modification strategy for plastic waste up-cycling.

## INTRODUCTION

With the ever-increasing amounts of plastics produced, recycling strategies, rather than landfilling and incineration, have to move in focus (1–3). Plastic waste represents not only a global pollution problem but also a carbon-rich, low-cost, globally available feedstock for industrial biotechnology (4, 5). Recently, an increasing number of studies using monomers of plastics derived from chemical or biological hydrolysis as fermenting substrate for value-added chemicals production has been reported, such as the production of aromatic derivatives from terephthalic acid (TPA, a monomer of Poly[ethylene terephthalate]) and the production of glycolic acid using ethylene glycol (EG, another monomer of Poly[ethylene terephthalate]) (6–8). The computed theoretical yields for industrially interesting products from well-known monomers imply their potential to replace the existing fossil-based synthesis routes for the same products (9). Therefore, making fermentation technology using plastics or monomers as raw materials would open up a broad spectrum of value-added products that can be produced by biotechnology, offering better end-of-life solutions for many unrecyclable plastics and plastic mixtures (10).

1,4-Butanediol (BDO) is a versatile intermediate for the chemical industry and widely used in many plastics production, including polybutylene terephthalate (PBT), poly butylene adipate-co-terephthalate (PBAT), poly (butylene succinate) (PBS), etc (11–14). Herein, construction of BDO valorization cell factory presents great significance for waste plastic up-recycling. In fact, P. putida possesses a completed the BDO degradation pathway, but it is inactive in normal culture conditions, leading to low cell growth when using BDO as the sole carbon source (15). In a previous study, Wing-Jin Li et al. has attempted to enhance BDO degradation capability of P. putida KT2440 by ALE method, and speculated the possible routes for BDO degradation in P. putida KT2440 through proteomics and comparative genomics (16). However, there are still many doubts that haven’t been revealed. For example, if succinate or succinyl-CoA routes are available in BDO degradation, if BDO degradation intermediates glycolyl-CoA can easily enter into the central metabolism network, and what is the limiting step during BDO degradation, etc. The unclear degradation pathway of BDO limits its further metabolism enhancement and downstream value-added chemicals synthetic pathway design through synthetic biology. Hence, further research focused on BDO metabolism is deserved to pave the way for plastic waste up-cycling.

In this study, P. putida NB10 mutant with enhanced growth rate on the medium with BDO as the sole carbon source was obtained via integration of ARTP and ALE. Further genome resequencing, transcriptome analysis and genetic engineering deeply revealed the metabolic mechanism behind BDO degradation in P. putida, offering an excellent chassis cell and a clear degradation pathway for plastic waste up-cycling.

## RESULTS AND DISCUSSION

### ARTP and ALE drives selection of P. putida KT2440 mutants with enhanced BDO utilization ability.

**FIG 1:**
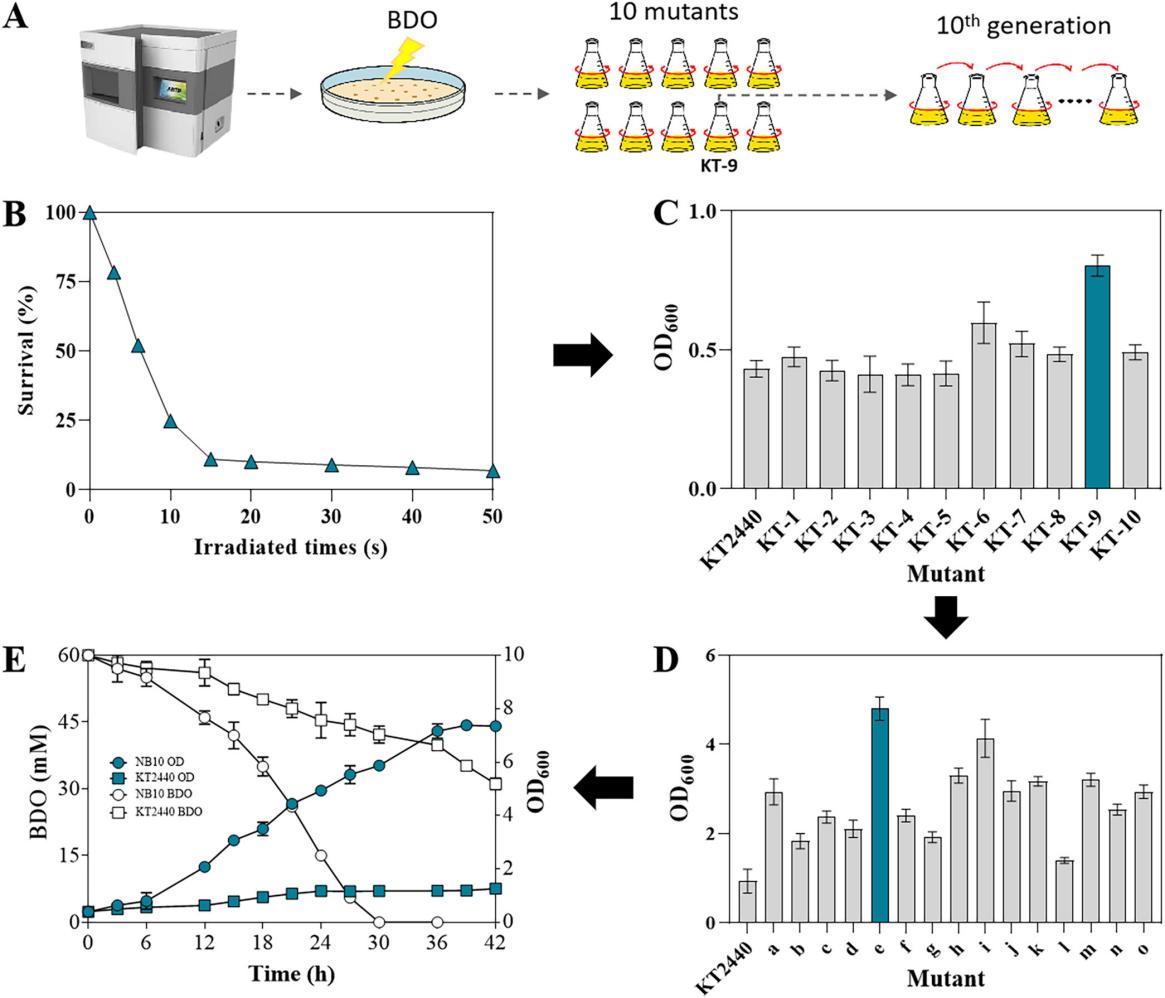
Screening and characterization of P. putida KT2440 mutant from ARTP and ALE. (A) Flow chart of ARTP and ALE screening; (B) Lethality of P. putida KT2440 at different irradiation times; (C) cell performance of the 10 mutants obtained after ARTP treatment; (D) ALE screening mutants from KT-9; (E) Comparison of cell growth and BDO degradation between P. putida KT2440 and P. putida NB10.

The generated plasma in ARTP system can seriously damage bacterial cells and thus induces mutations, while ALE is an innovative approach that mimics natural selection to obtain desired microbes, hence, the combination of ARTP mutagenesis tool and ALE method is widely used for strain evolution and discovery of novel metabolic pathways or enzymes (17, 18). In this study, ARTP and ALE were used to accelerate P. putida KT2440 evolution for screening of mutants with enhanced BDO utilization ability (Fig. 1A). The cell fatality rate was first evaluated by treating the dilute cells with different times (0, 3, 6, 10, 15, 20, 30, 40, 50 s) in helium plasma. After 20 s treatment, the survival rate of P. putida KT2440 was 8.9 ± 0.8% (<10%) (Fig. 1B). Accordingly, a lethality rate of 90% was considered appropriate (19). Therefore, 20 s was selected as the optimal exposure time to generate mutations in P. putida KT2440. In a primary screen, 10 mutants were collected from M9 agar with 1.8 g/L of BDO as the sole carbon source. Among the ARTP-mutated strains, the mutant (KT-9) exhibited the best BDO degradation capability in the second liquid fermentation, as 86% improvement in cell growth was observed compared with the wide type (Fig. 1C). Hence, the mutant KT-9 was selected for further ALE mutation.

After 10 generations of ALE, a P. putida KT2440 mutant e, exhibiting the fast growth performance cultivated on BDO medium, as up to 4.7 of OD_600_ after 24 h cultivation was detected, displaying almost 5 times higher than the parental P. putida KT2440 (Fig. 1D). The mutant e was renamed as P. putida NB10 and has been stored in China Typical Culture Preservation Center (CCTCC), the deposit number is CCTCC NO: M 2021482. P. putida NB10 grew fast in the medium with BDO as the sole carbon source, and the maximum OD_600_ of 7.49 was reached after 42 h cultivation (Fig. 1E). However, no significant growth was observed in the parental P. putida KT2440, and only an OD_600_ of 1.41 could be reached after 42 h cultivation. Consistent with the cell growth, BDO consumption also displayed a great difference between P. putida KT2440 and P. putida NB10 (Fig. 1E). 60 mM BDO could be completely consumed by P. putida NB10 after 30 h fermentation, while only about 30 mM BDO has been utilized by P. putida KT2440.

Further evaluations by using different concentrations of BDO also confirmed that the BDO degradation pathway was indeed enhanced in P. putida NB10. After 42 h cultivation in BDO contained M9 medium, an increasingly apparent in cell growth enhancement of NB10 was noted (Fig. S1). And a relatively high cell mass could be obtained from BDO in the range of 120~300 mM. When BDO was increased up to 500 mM, an obvious growth inhibition was caused by the intermediate cytotoxicity from BDO, even though no cell growth was found once BDO concentration increased to 1000 mM. It was reported that some intermediates, especially aldehydes during BDO degradation instead BDO itself is cytotoxic, the mutant NB10 might possess a better tolerance or an enhanced pathway to timely degrade these toxic intermediates. Indeed, the toxicity test showed that P. putida NB10 exhibited a better tolerance against aldehydes (glycolaldehyde, glyoxylate, and 4-hudroxybutyraldehyde) than P. putida KT2440 (Fig. S2), However, it is still limited to disposing too much high BDO concentration. Further mechanism analysis will provide more rational strategies using synthetic technology to improve BDO degradation efficiency.

### Genome-wide analysis of ARTP-ALE integration induced SNP/InDel mutations in P. putida NB10.

**FIG 2:**
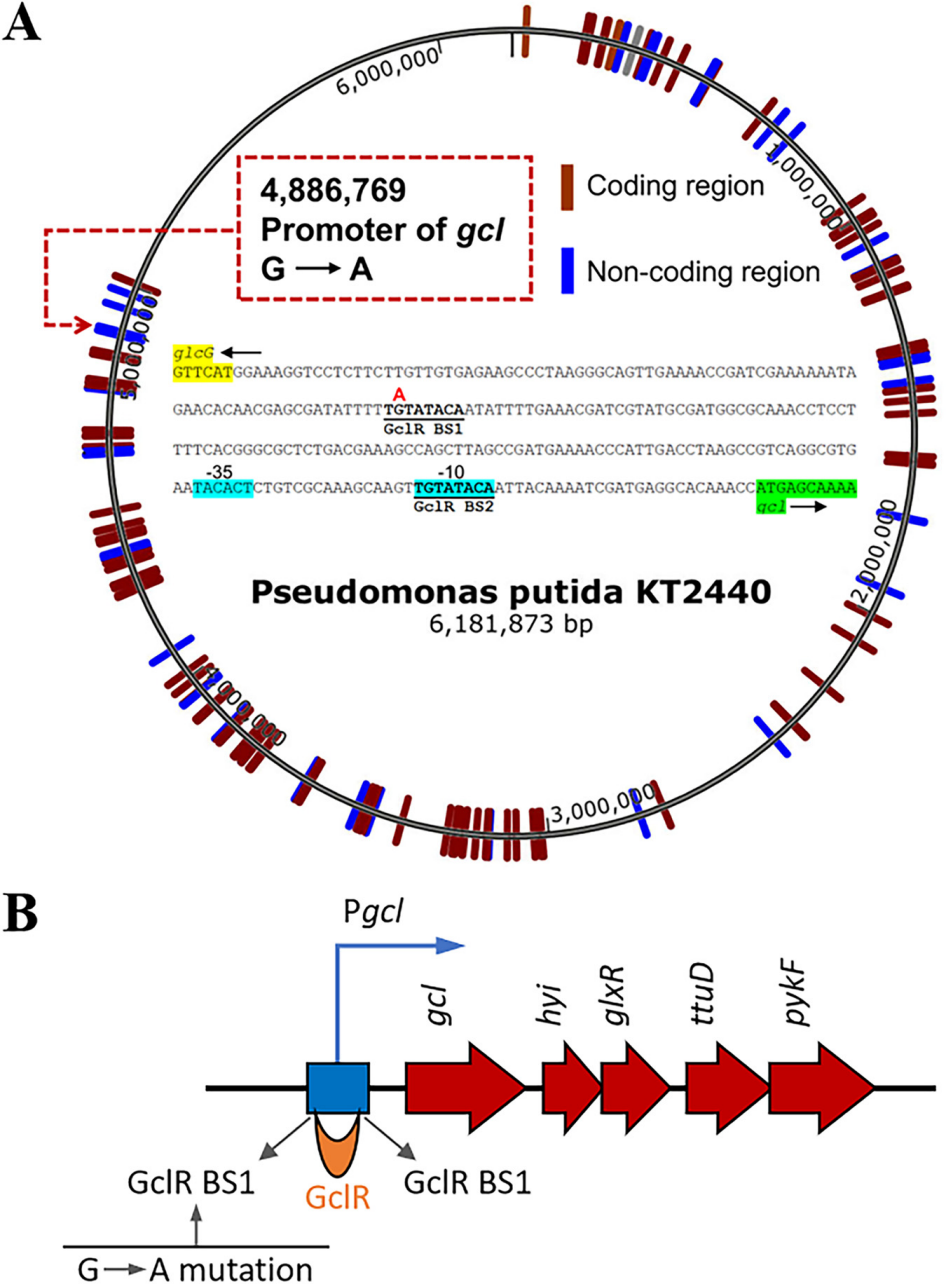
Genome-wide analysis of SNP/InDel mutations in P. putida NB10. (A) SNP/InDel analysis of the mutant NB10 mapped to the KT2440 reference genome (Ae015451.2), the red line represented the SNP/InDel mutations that occurred in the coding region, the blue line represented the SNP/InDel mutations occurred in the noncoding region; (B) Gene organization in GCL pathway and a mutation occurred in BS1.

To gain insights into the phenotypic changes that occurred in the evolved strain NB10, genomic comparison between P. putida NB10 and P. putida KT2440 was carried out. It was found that 160 mutations occurred, including SNPs and InDel in the genome of P. putida NB10 (Table S1), with 38.8% of these mutations were silent or intergenic, and 61.2% of mutations occurred into functional genes (Fig. 2A). After analysis of each mutation site one-by-one, three mutations occurred in gene *rpoB*, encoding RNA polymerase β-subunit, was selected for further analysis (Table S1), sue to that *rpoB* mutation was reported could alter the expression of many genes under its transcriptional control (20). Adoption of robust microbial chassis with great tolerance to harsh culture conditions or inhibitors through the screen of *rpoB* mutation has also been applied (21). The mutations of *ropB* in NB10 may also improve BDO utilization or tolerance to aldehydes.

In addition, one mutation occurred in the promoter region of GCL pathway (*gcl*-*hyi*-glxR-*ttuD*-*pyk*) gene cluster (22), which was reported to play a significant role in BDO metabolism. The native GCL pathway (a gene cluster consisting of *gcl*, *hyi*, *glxR*, *ttuD*, and *pyk*) is inactive, since the binding of GclR (glyoxylate carboligase repressor) in the promoter region. In NB10, a codon mispairing (G to A) at the position 4886769 in this promoter disrupted the binding site (BS1) of GclR to the *gcl* promoter (Fig. 2B) (23, 24), releasing a putative promoter so that resulted in significant gene transcription increase in GCL pathway. In a previous report, BDO metabolizes intermediate glycolyl-CoA would be reduced to glycolaldehyde and successively oxidized to glycolate and glyoxylate for cell growth (16, 25, 26). While glyoxylate can either be metabolized into malate by ligation acetyl-CoA or converted into isocitrate by ligation to succinate, however, the performance of both possible pathways depends on sufficient acetyl-CoA supply (22). Otherwise, another pathway for accumulated glyoxylate transformation into central metabolic pathway was needed. Here, the flux change of GCL pathway would directly influence on glyoxylate metabolism to acetyl-CoA. Further transcription analysis of the gene cluster in GCL pathway described in the next section result will confirm this guess.

### Transcriptome analysis of the NB10 mutant grow on BDO medium by RNA-seq.

Transcriptome of NB10 and KT2440 grow with BDO as sole carbon source were investigated by RNA-seq analysis. The result exhibited 1722 differentially expressed genes (DEGs) performed at least 2-fold upregulated or downregulated in expression levels in P. putida NB10, in which, 991 genes were upregulated and 731 genes were downregulated (Fig. S3). The transcription of *rpoB* was decreased by 1.6 times. However, an expression of the mutant *rpoB* of P. putida NB10 in P. putida KT2440 demonstrated no visible difference of cell growth with BDO as sole carbon source (Fig. S4), indicating the negligible influence on BDO degradation caused by the mutations occurred in *rpoB*.

**FIG 3:**
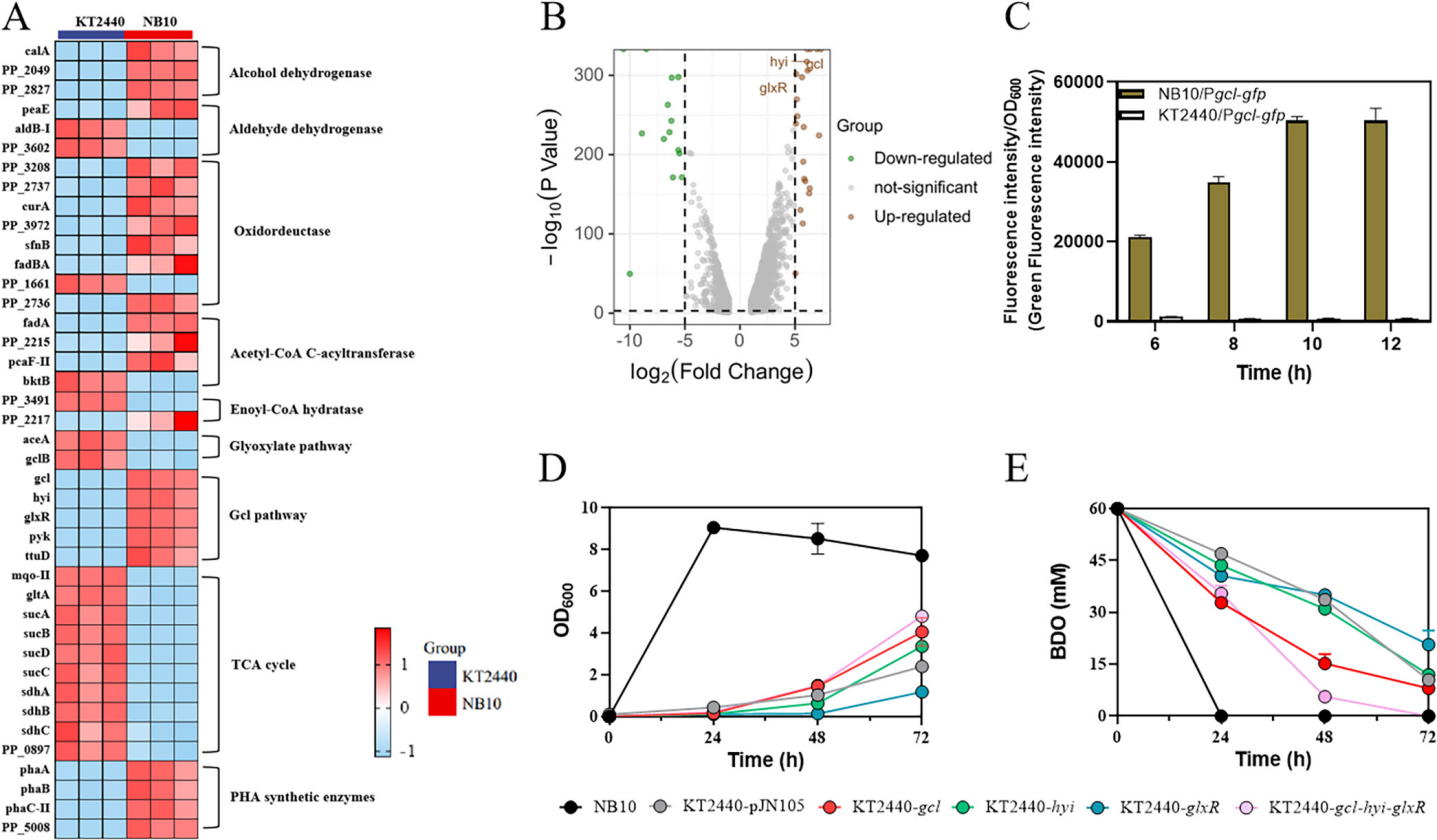
Transcriptome analysis and functional genes verification. (B) Hierarchical clustering analysis of genes markedly differentially transcriptional in response to BDO degradation; (A) Differential transcriptional analysis of intimate genes involved BDO degradation between P. putida NB10 and P. putida KT2440; (C) Strength detection of the *gcl* promoter from NB10 and KT2440 using pPROBE-*gfp* as report plasmid. (D and E) The influence of key genes (*gcl*, *hyi*, *glxR*) expression in GCL pathway on cell growth and BDO degradation.

Further analysis of the transcription of genes in GCL pathway showed, 2^7.34, 2^6.07, 2^5.11, 2^3.25 and 2^3.21-fold transcription improved by genes coding for glyoxylate carboligase (*gcl*), hydroxypyruvate isomerase (*hyi*), 2-hydroxy-3-oxopropionate reductase (*glxR*), pyruvate kinase (*pyk*) and glycerate kinase (*ttuD*) were detected. The mutation in the promoter of GCL gene cluster might do help in increasing these genes transcription; besides, the enhancement of GCL pathway also implies the significance of GCL pathway in BDO degradation. To accurately evaluate the difference between promoter activity of GCL cluster promoter between NB10 and KT2440, plasmids of NB10/P*gcl*-*gfp* and KT2440/P*gcl*-*gfp* carried the GCL promoter from NB10 and KT2440 were constructed and transformed into KT2440. As shown in Fig. 3C, the intensity of green fluorescence regulated by the mutated promoter from NB10 exhibited 17~74 times higher than the wild-type promoter from KT2440, indicating the positive effect of the mutated promoter on GCL pathway regulation. Correspondingly, overexpression of the key genes in GCL pathway has also confirmed the positive role of GCL pathway in BDO degradation. Fig. 3D and 3E illustrated the overexpression of the GCL pathway genes, especially the coexpression of *gcl*, *hyi* and *glxR* significantly enhanced BDO degradation by KT2440.

Previous speculation that BDO might be mostly converted into 4-hydroxy-3-keto-butyryl-CoA, then splitting into acetyl-CoA and glycolyl-CoA, acetyl-CoA is the central metabolites for many metabolic pathways, while glycolyl-CoA may be converted into glycolate and subsequently metabolized through the native pathway in P. putida KT2440 (16). Indeed, the transcription of most enzymes involved in this pathway was significantly upregulated, especially the enzyme of alcohol dehydrogenase (*calA*, PP_2049, PP_2827), aldehyde dehydrogenase (*peaE*), oxidoreductase (PP_3208, PP_2737, *fadBA*), acetyl-CoA C-acyltransferase (PP_2215), enoyl-CoA hydratase (PP_2217), acetyl-CoA C-acyltransferase (*fadA*, PP_2215, *pcaF-II*), and especially the enzymes in GCL pathway (Fig. 3A). Hence, this pathway and enhances GCL module do improve the BDO degradation. However, BDO utilization of these engineered strains was still inferior to NB10, on one side, more evolutions in the aspect of such as alcohols or intermediate (mainly aldehydes) tolerance, global regulation and carbon flux improvement might cause a comprehensive enhancement for BDO degradation by NB10.

Except for the genes related to BDO degradation, significant transcription increase of the key genes involved in PHB synthesis, including *phaA*, *phaB*, *phaC-II*, and *phi* (PP_5008) were also improved. Metabolites analysis also verified that PHB accumulation was increased from 3.54% in KT2440 to 8.44% in NB10 in dry cell weight (Fig. S3). It has been confirmed that PHB mobilization in bacteria can benefit their host for survival under stress conditions (27). The enhanced PHB accumulation should be a benefit for cells to survive after the strong plasma treatment, or intensify the fragile cell tolerate the cytotoxic metabolic intermediates during BDO degradation.

Moreover, a glance of the downregulated genes found that the transcription of all enzymes that participate in TCA cycle were decreased in different levels (Fig. 3A), implying less carbon atom entered into TCA cycle after the enhancement of BDO degradation, and the speculation that BDO can be transferred to succinate or succinate-CoA and then enter into central metabolic pathway might do a negligible role for BDO degradation in mutant NB10.

### BDO metabolism in the P. putida KT2440 and P. putida NB10.

To summarize, BDO degradation pathway is quite different from its synthetic pathway, where the TCA cycle intermediate succinyl-CoA is the initial compound for desired product synthesis (28). For BDO degradation, the reverse pathway from BDO to succinyl-CoA was downregulated in the mutant NB10, indicating its futility during BDO metabolism. Also, no upregulated transcriptional of genes were detected in the supposed pathway from BDO to succinate was detected. On the contrary, the transcription of genes in TCA cycle was universally downregulated, indicating that the compounds in TCA cycle might be not the intermediate during BDO degradation.

**FIG 4:**
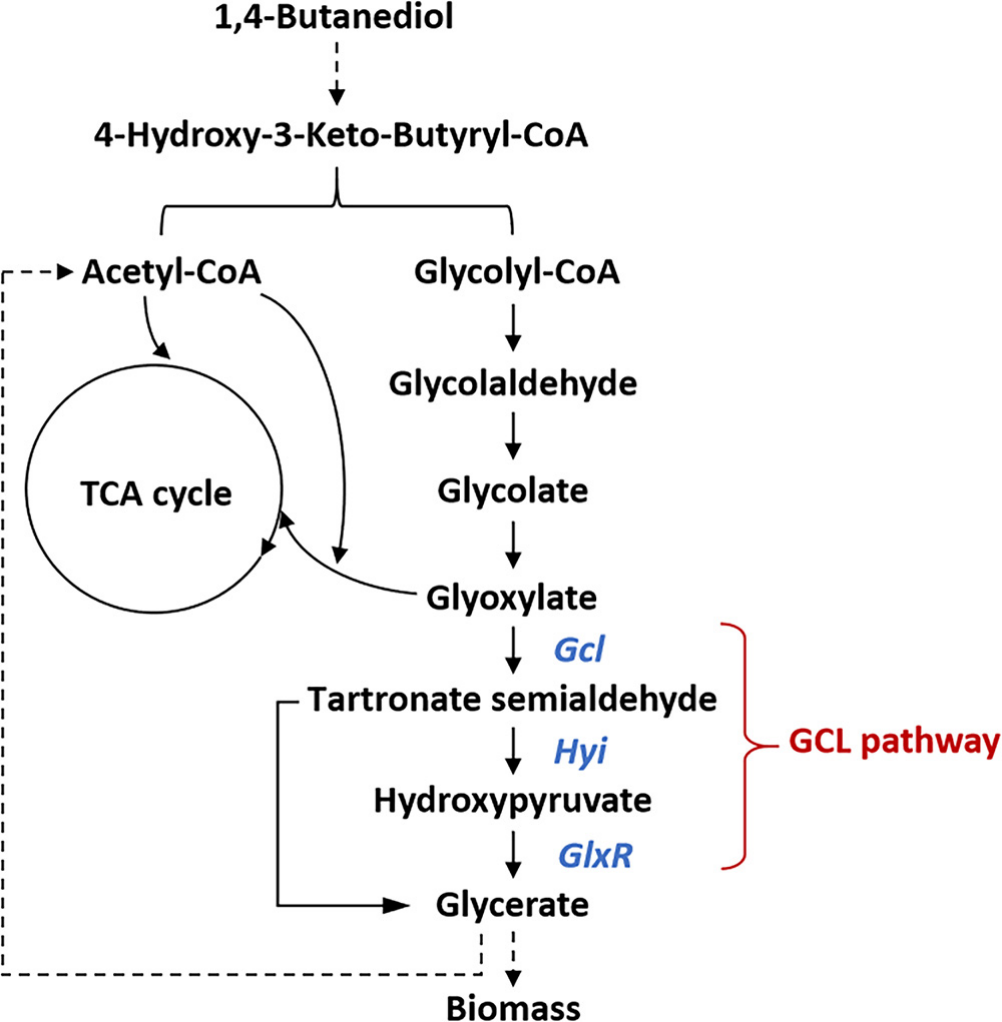
BDO metabolic pathway mapping in P. putida NB10.

Comprehensive consideration of the transcription of genes in the pathway from BDO to glycolyl-CoA and subsequent to GCL pathway, BDO degradation pathway was confirmed to be composed of β-oxidation, GCL pathway, glyoxylate cycle and gluconeogenesis pathway (Fig. 4). First, BDO is oxidized to 4-hydroxybutyrate by a series of alcohol/aldehyde dehydrogenase and oxidoreductase, then transferred to 4-hydroxy-3-keto-butyryl-CoA and subsequently split into acetyl-CoA and glycolyl-CoA; Glycolyl-CoA can be easily transferred to glyoxylate, which can either into glyoxylate pathway by ligation to acetyl-CoA or through the TCA cycle initiated by ligation to succinate (22). However, the decarboxylic reaction during TCA cycle compels no glyoxylate can be used for cell growth by ligation to succinate (29). In terms of ligation to acetyl-CoA, two molecules of acetyl-CoA were needed to hold glyoxylate pathway running. Therefore, concentration imbalance between acetyl-CoA and glyoxylate led to relatively worse cell growth and BDO degradation by KT2440. In NB10, the activation of GCL pathway provided glyoxylate another metabolic pathway, extra acetyl-CoA can be synthesized through GCL and gluconeogenesis pathway. In conclusion, acetyl-CoA generated from BDO can enter TCA cycle directly or can be used for PHB synthesis, while the glycolyl-CoA generated will mainly go through GCL pathway and gluconeogenesis pathway, so that to maintain cell growth, and the extra carbon flux from glycolyl-CoA will be further transformed into acetyl-CoA, which will be metabolized as described above.

### Conclusion.

Integration of ARTP and ALE was proved to be an efficient strategy to screen robust and competent microorganism chassis for stubborn substrate degradation. The mutant of NB10 achieved from ARTP and ALE exhibited great BDO degradation capability and enhanced BDO tolerance. Deep molecular mechanism revelation of BDO degradation enabled more possibility for enhancing DBO degradation via genetic engineering. The fast development of synthetic biotechnology and fermentation engineering technology will boost more promising biological pathways from more plastic depolymerization products to high-value bio-based compounds, and the utilization of recycled waste plastic for next generation fermentation substrate might be achieved in the near future.

## MATERIALS AND METHODS

### Microorganism, media, and cultivation conditions.

All strains used in this work were listed in Table 1. Wild-type and engineered strains were cultivated in sterile seed medium LB (5 g/L yeast extract, 10 g/L tryptone, 10 g/L NaCl), incubated at 30°C, 200 rpm for 12 h. Antibiotics were added where appropriate to following final concentrations: kanamycin (50 mg/L), gentamicin (30 mg/L).

**TABLE 1:** Microorganisms and plasmids used in this study

Strain or plasmid	Relevant characteristics	Source
Strain		
P. putida KT2440	Wild-type (WT) strain designated KT2440	(32)
P. putida NB10	KT2440 mutant obtained from ARTP and adaptive laboratory evolution	This study
KT2440-pJN105	WT strain carries plasmid pJN105 as a control	This study
KT2440-pJN105-*rpoB*	KT2440 *rpoB* from NB10	This study
KT2440-pJN105-*gcl*	KT2440 expressed *gcl* from NB10	This study
KT2440-pJN105-*hyi*	KT2440 expressed *hyi* from NB10	This study
KT2440-pJN105-*glxR*	KT2440 expressed *glxR* from NB10	This study
KT2440-pJN105-*gcl*-*hyi*-*glxR*	KT2440 expressed gene cluster of GCL pathway *(gcl-hyi-glxR)* from NB10	This study
Plasmid		
pJN105	*araC*-pBAD cassette cloned in pBBR1MCS-5	(33)
pPROBE-*gfp*	pBBR1 ori, Kmr, broad-host range expression vector	(34)
KT2440/P*gcl*-*gfp*	*gcl* promoter from KT2440 cloned into pPROBE-*gfp*	This study
NB10/P*gcl*-*gfp*	*gcl* promoter from NB10 cloned into pPROBE-*gfp*	This study

Shake flask experiments were performed using M9 minimal media (Sinopharm Chemical Reagent Co., Ltd.) containing specified concentration of BDO (20 mM for agar plates and liquid fermentation in the experiment of ARTP and ALE, 60 mM and more concentrations for shake flask fermentation), 6.78 g/L Na_2_HPO_3_, 3 g/L KH_2_PO_3_, 0.5 g/L NaCl, 1 g/L NH_4_Cl, 2 mM MgSO_4_, 100 μM CaCl_2_, and 40 μM FeSO_4_·7H_2_O, incubated at 30°C, 200 rpm.

### Mutagenesis by ARTP and ALE.

P. putida KT2440 was connected at logarithmic stage from M9 minimal media with glucose as carbon source, the harvested cell pellets was then washed and diluted to OD_600_ = 1 by 0.85% saline. Afterwards, 10 μL of the bacterial solution was evenly coated on a sterile metal plate and dried by sterile air. Then the metal plate was placed in ARTP device. the ARTP device used a plasma generator to achieve a stable glow discharge of pure helium through a metal electrode structure. The operating parameters of the machine were set accordingly to Dong et al. (30). The metal plate was exposed to helium plasma jet for 0, 3, 6, 10, 15, 20, 30, 40, 50 s, respectively. The treated cells were serially diluted to an appropriate concentration by sterile saline (0.85%) and inoculated onto LB agar to determine cell survival rates. The lethality rate was calculated as follows: lethality rate = (1 - N1/N0) × 100%, where N0 is the colony number of the control (0 s treatment) and N1 is the colony number of the mutants. Then, the mutant cells treated with an appropriate time was diluted and inoculated onto M9 agar with 20 mM BDO as the sole carbon source, the plates were cultivated at 30°C for 24 h.

Subsequently, the fast grow mutant colony was transferred in a fresh M9 liquid medium, cells were connected at logarithmic stage and the diluted broth was inoculated onto M9 agar again. After 10 times alternate culture and screening on M9 agar and liquid medium, the fast grow colony was selected for further evaluation.

### Molecular work.

Some genes expression was carried out to confirm BDO metabolic pathway. The plasmids construction was performed by using One Step Cloning kit (Vazyme, China). DNA modifying enzymes were purchased from Vazyme (China). The primer pairs used in this study were listed in Table 2. Clonal DNA sequences were amplified using the 2 × Phanta Flash Master Mix (Vazyme, China).

**TABLE 2:** Primes pairs used in this study

Primer pair	Sequence (5′-3′)
*rpoB*-pJN-F	TGGGCTAGCGAATTCATGGCTTACTCATACACTGAGA
*rpoB*-pJN-R	TAGGGCGAATTGGAGCTCTTATTCGGTTTCCAGATCG
*gcl*-pJN-F	TGGGCTAGCGAATTCATGAGCAAAATGAGAGCAATCGA
*gcl*-pJN-R	TATAGGGCGAATTGGAGCTCTCAGTCCAGCAGCGAGATG
*hyi*-pJN-F	TGGGCTAGCGAATTCATGCCTCGCTTCGCTGCCAAC
*hyi*-pJN-R	TAGGGCGAATTGGAGCTCTCAGATTGCGTTGTGGGT
*glxR*-pJN-F	CCCGTTTTTTTGGGCTAGCGAATTCATGGCTAAAATCGGTTTCATC
*glxR*-pJN-R	CTATAGGGCGAATTGGAGCTCTTATTTGTCGTCGCGGATCGAG
P*gcl*_KT2440_-F	GGTACCAAGGCCTGAGCTCGGTTTGTGCCTCATCGATTTTG
P*gcl*_KT2440_-R	GGGGATCGGAAGCTGAATTCGGAAAGGTCCTCTTCTTGTT
P*gcl*_NB10_-F	GGTACCAAGGCCTGAGCTCGGTTTGTGCCTCATCGATTTTG
P*gcl*_NB10_-R	GGGGATCGGAAGCTGAATTCGGAAAGGTCCTCTTCTTGTT

For gene expression, plasmid pJN105 was used to construct overexpression plasmids. The primers were designed according to the gene sequences of *gcl*, *hyi*, *glxR*, *rpoB* and the gene cluster of GCL pathway (*gcl-hyi-glxR*) from P. putida NB10 (Table 2), the upstream and downstream primers were, respectively, introduced into the homology arms of the *Sac* I and EcoR I restriction sites in the pJN105 plasmid. The above plasmids were transformed into P. putida KT2440 by electroporation and screened on plate containing 30 mg/L of gentamicin.

To detect the strength of the *gcl* mutated promoter, the intergenic region upstream of the *gcl* coding region from P. putida KT2440 and P. putida NB10 was cloned into the pPROBE-*gfp* vector between the restriction sites of EcoR I and *Sac* I, to get plasmids KT2440/P*gcl*-*gfp* and NB10/P*gcl*-*gfp*. The obtained expression vector was transformed into P. putida KT2440 by electroporation and screened on plate containing 50 mg/L of kanamycin.

### Analytical methods.

**(i) Determination of cell mass**. Cell growth was determined by OD_600_. All samples were diluted by fermentation medium to an optical density of 0.2 to 0.8 for measurement.

**(ii) BDO determination.** 0.1 mL of fermentation broth was added into 1 mL acetonitrile, then 0.2 g anhydrous sodium sulfate was added to the mixture, mixed, and incubated for 1 min. After the final step of filtration by 0.45 μm filter, the filtrate was used for gas chromatography (GC) analysis. BDO determination was performed with a standard GC device (GC9720PLUS, Fuli instruments, China) equipped with a HP Innowax column (15 m × 0.25 mm × 1m) and a flame ionization detector (FID). An oven ramp cycle was employed as follows: 140°C for 5 min, then increasing by 4°C/min to 230°C, keeping at 230°C for 8 min. A 100:1 split was used with helium as the carrier gas and an inlet temperature of 260°C.

### Toxicity test of aldehydes involved in BDO degradation.

Overnight cultures of P. putida KT2440 and P. putida NB10 grown in LB were subsequently concentrated by centrifugation and inoculated into M9 medium containing 20 mM glucose and various concentrations of aldehydes with the starting OD_600_ of 0.05 to 0.1, the cells were cultivated as described above for 48 h, absorbance readings were taken every 12 h.

### Genome sequencing and transcriptome analysis.

Both P. putida KT2440 and the mutant strain NB10 were cultured in a shake flask with M9 basic medium containing 60 mM BDO (three groups). The midexponentially growing (at around 36 h) cell pellets were collected and quick-frozen by liquid nitrogen. The samples were used for genome sequencing and SNP/InDel (single nucleotide polymorphism/insertion and deletion polymorphism) analysis by Magigene (China). To identify single nucleotide polymorphisms (SNPs), the trimmed reads of mutant NB10 were mapped to the KT2440 reference genome (Ae015451.2) using BWA with default parameters. SAM file-to BAM file conversion was performed using SAMtools. SNPs and insertions or deletions (indels) were called using mpileup from samtools and bcftools. SNP sites were visualized by SnapGene.

Transcriptome analysis were also taken by Magigene (China). DEGs with false discovery rate (FDR) ≤ 0.001 and fold change ratio larger than 2 were chosen for gene ontology (GO) functional enrichment analysis and KEGG pathway analysis. Only the genes exhibiting the *P* value < 0.05 and Q-value < 0.05 were considered differentially expressed (31).

### Promoter activity analysis.

To analyze the correlation between promoter and expression intensity of enhanced green fluorescent protein. Synergy HTX multimode reader (BioTek, Winooski, VT, USA) was used to analyze and calculate the total GFP fluorescence intensity at 490 nm excitation light wavelength and 516 nm emission light. The culture medium was measured by a pipette gun and 200 μL was placed in a 96-well black polystyrene plate (Fisher Scientific, Pittsburgh, PA, USA) to measure the fluorescence intensity.

### Polyhydroxybutyrate (PHB) production assay.

Weighing a certain amount of freeze-dried cell pellet (about 50 mg) into the esterification tube, then 2 mL of chloroform and 1 mL of esterification reaction solution (1 g benzoic acid dissolved in 100 mL methanol and 10 mL concentrated H_2_SO_4_) was added into the tube. After cooling at room temperature, add 1 mL of deionized water into the reaction system, fully mixed the system by a whirlpool mixer, then stored the system at 4°C overnight until the lower organic phase become clarified. Then, 1 mL of the lower organic phase was used for GC analysis.

Measurement was performed using standard GC equipment (GC9720PLUS, Fuli instruments, China) equipped with a HP Innowax column (15 m × 0.25 mm × 1m) and a flame ionization detector (FID). An oven ramp cycle was employed as follows, 140°C for 5 min, then increasing by 4°C/min to 230°C, keeping at 230°C for 8 min. A 100:1 split was used with helium as the carrier gas and an inlet temperature of 260°C.
