# Supplementary material for: Integration of ARTP Mutation and Adaptive Laboratory Evolution to Reveal 1,4-Butanediol Degradation in Pseudomonas putida KT2440
Source: Microbiol Spectr. 2023 Apr 17;11(3):e04988-22. doi: 10.1128/spectrum.04988-22 (PMC10269461; doi:10.1128/spectrum.04988-22)
Supplement: Supplemental file 1 — Supplemental material. Download spectrum.04988-22-s0001.pdf, PDF file, 0.4 MB [file spectrum.04988-22-s0001.pdf]

Table S1. Genetic codon mutation occurred in *P. putida* NB10.

Mutation	Position	KT2440	NB10	Mutated gene	Function
1	40912	C	A	oprP	porin P
2	185951	G	A	PP_0162	ECF family RNA polymerase sigma-70 factor
3	195559	C	A	PP_0168	putative surface adhesion protein
4	195634	C	T	PP_0168	putative surface adhesion protein
5	229937	G	A	PP_0178	efflux pump
6	233051	C	T	PP_0180	cytochrome c family protein
7	259778	C	A	PP_0207	nitrate ABC transporter substrate-binding protein
8	277467	G	A	PP_0224	DszC family monooxygenase
9	278203	C	A	PP_0224	DszC family monooxygenase
10	278742	TAA	TAAA	Non-coding regions	-
11	307865	GC	GCC	Miscellaneous	pck (pseudogene)
12	336124	AT	ATT	PP_0278	hypothetical protein
13	337116	G	A	PP_0279	hypothetical protein
14	353067	A	G	Non-coding regions	-
15	381867	C	T	PP_0317	methyl-accepting chemotaxis transducer
16	425580	C	T	PP_0350	ferrichrome-iron receptor
17	499203	A	G	Non-coding regions	-
18	508255	G	T	estP	esterase EstP
19	540242	G	T	rpoB	RNA polymerase β-subunit
20	540563	C	T	rpoB	RNA polymerase β-subunit
21	540608	A	G	rpoB	RNA polymerase β-subunit
22	662322	G	A	PP_0569	MATE efflux family protein
23	699722	A	G	Non-coding regions	-
24	748798	G	A	Non-coding regions	-
25	965647	G	A	ptxC	phosphonate transport system permease PtxC
26	1005267	G	A	PP_0867	FecA-like outer membrane receptor

27	1013729	G	A	potF-1	putrescine-binding protein
28	1037005	G	A	PP_0896	carbon-nitrogen hydrolase family protein
29	1070246	TGA	TGAGA	Non-coding regions	-
30	1126645	ACC	ACCC	Non-coding regions	-
31	1130831	G	A	PP_0991	hypothetical protein
32	1131444	G	A	Non-coding regions	-
33	1131692	C	T	PP_0992	hypothetical protein
34	1134816	C	A	gltS	glutamate:sodium symporter
35	1155532	G	A	PP_1013	sensor histidine kinase
36	1156102	G	T	PP_1013	sensor histidine kinase
37	1195271	C	T	uxpA	lipoprotein UxpA
38	1200188	G	T	gspF	protein secretion protein
39	1333855	G	A	PP_1159	membrane protein
40	1341460	G	A	PP_1166	DMT superfamily permease
41	1341753	G	A	PP_1166	DMT superfamily permease
42	1356854	G	A	PP_1182	sensor histidine kinase
43	1371747	G	C	Non-coding regions	-
44	1419548	GC	GCC	Non-coding regions	-
45	1438537	G	T	yveA	aspartate-proton symporter
46	1438901	G	A	yveA	aspartate-proton symporter
47	1465418	T	C	algX	alginate biosynthesis protein AlgX
48	1497836	C	T	PP_1310	hypothetical protein
49	1600960	C	A	bglX	beta-D-glucoside glucohydrolase
50	1618357	T	C	PP_1418	tricarboxylate transport protein TctC
51	1619778	G	A	opdH	tricarboxylate-specific outer membrane porin
52	1752445	G	T	Non-coding regions	-
53	1754370	C	T	Non-coding	-

				regions	
54	1932638	GCC	GCCC	Non-coding regions	-
55	2005413	G	T	PP_1788	hypothetical protein
56	2075756	G	T	PP_5479	LysR family transcriptional regulator
57	2210975	CCGCGC	CCGC	PP_1954	hypothetical protein
58	2328108	G	A	PP_2046	LysR family transcriptional regulator
59	2331209	A	T	PP_2048	acyl-CoA dehydrogenase
60	2388895	C	T	Non-coding regions	-
61	2388951	T	A	Non-coding regions	-
62	2706983	G	C	PP_2369	leucine-rich repeat-containing protein
63	2766503	C	T	Non-coding regions	-
64	3011425	G	A	bcsG	endoglucanase
65	3015377	C	A	bcsA	cellulose synthase and translocator subunit
66	3031010	C	T	mgtA	ATP-dependent magnesium transporter
67	3074167	G	A	PP_2684	membrane protein
68	3096075	C	T	PP_2704	M20/M25/M40 family peptidase
69	3139381	C	A	Non-coding regions	-
70	3145559	C	T	PP_2760	ribose ABC transporter permease
71	3146131	C	T	PP_2761	ribose ABC transporter permease
72	3172200	G	T	PP_2784	short-chain dehydrogenase/reductase family oxidoreductase
73	3200236	C	T	PP_2807	hypothetical protein
74	3200692	C	T	PP_2808	oxidoreductase
75	3218367	C	T	oprJ	outer membrane protein OprJ
76	3224649	C	T	mexT	transcriptional regulator MexT
77	3243740	C	T	PP_2839	hypothetical protein

78	3357071	C	T	PP_2953	alcohol dehydrogenase
79	3429798	C	T	Non-coding regions	-
80	3444478	C	A	PP_3062	tail length determination protein
81	3444771	C	T	gpU	tail formation protein
82	3453379	G	A	PP_3069	outer membrane autotransporter
83	3472581	C	T	Non-coding regions	-
84	3600180	G	A	PP_3176	MFS transporter
85	3613738	C	T	Non-coding regions	-
86	3736410	C	T	PP_3302	RND family transporter
87	3738373	G	A	PP_3303	3-oxoacyl-ACP synthase
88	3741130	C	T	PP_3305	TerC family membrane protein
89	3762893	T	A	PP_3326	hypothetical protein
90	3764536	G	A	Non-coding regions	-
91	3783152	C	T	nikD	nickel ABC transporter ATP-binding protein
92	3787091	C	T	mhpT	3-(3-hydroxy-phenyl)propionate transporter MhpT
93	3831512	G	A	PP_3384	gluconate 2-dehydrogenase gamma subunit
94	3845703	CGG	CGGG	Non-coding regions	-
95	3903560	GGCGCGC	GGCGC	ilvA-I	threonine deaminase
96	3909102	C	T	PP_3450	TPR repeat-containing protein
97	3909249	G	A	PP_3450	TPR repeat-containing protein
98	3951159	T	C	Non-coding regions	-
99	3951161	A	T	Non-coding regions	-
100	3955285	C	T	mnxG	manganese-oxidizing multicopper oxidase
101	3984559	C	T	PP_3512	membrane protein
102	4011003	G	A	pobR	transcriptional regulator
103	4073619	C	T	Non-coding	-

				regions	
104	4245833	G	T	alr	alanine racemase
105	4249298	C	T	PP_3724	acyl-CoA synthetase
106	4267798	G	A	galP-IV	porin-like protein
107	4275934	G	A	glcE	glycolate oxidase FAD-binding subunit
108	4313572	G	A	PP_3785	hypothetical protein
109	4329741	G	A	PP_3801	cation ABC transporter substrate-binding protein
110	4339638	A	C	ribAB-II	3,4-dihydroxy-2-butanone-4-p hosphate synthase
111	4348705	A	G	Non-coding regions	-
112	4377382	CTTTTTT TTTT	CTTTT TTTTT TTTTT	PP_3852	BNR domain-containing protein
113	4384007	G	A	PP_3859	hypothetical protein
114	4386541	A	G	PP_3863	tail protein
115	4405947	A	G	PP_3887	hypothetical protein
116	4406441	A	G	PP_3888	hypothetical protein
117	4446337	G	A	nicD	N-formylmaleamate deformylase
118	4586030	C	T	Non-coding regions	-
119	4586031	T	C	Non-coding regions	-
120	4586033	CGG	CGGG	Non-coding regions	-
121	4586056	AC	ACC	Non-coding regions	-
122	4604083	G	T	Miscellaneous	
123	4604208	C	A	tssE2	Protein secretion/export apparatus
124	4629536	C	T	PP_4094	hypothetical protein
125	4643760	C	T	PP_4108	4-aminobutyrate aminotransferase
126	4740816	T	G	Non-coding regions	-
127	4740819	T	C	Non-coding regions	-

128	4740820	G	T	Non-coding regions	-
129	4741229	A	C	Miscellaneous	-
130	4741231	C	G	Miscellaneous	-
131	4741234	C	T	Miscellaneous	-
132	4741236	G	T	Miscellaneous	-
133	4741237	C	G	Miscellaneous	-
134	4741239	A	G	Miscellaneous	-
135	4741243	G	T	Miscellaneous	-
136	4741244	C	G	Miscellaneous	-
137	4741245	A	C	Miscellaneous	-
138	4741255	C	T	Miscellaneous	-
139	4741257	A	C	Miscellaneous	-
140	4741260	C	G	Non-coding regions	-
141	4741261	A	C	Non-coding regions	-
142	4741263	A	C	Non-coding regions	-
143	4741272	A	G	Non-coding regions	-
144	4741274	A	C	Non-coding regions	-
145	4741276	G	C	Non-coding regions	-
146	4741278	C	T	Non-coding regions	-
147	4741289	C	G	Non-coding regions	-
148	4741292	C	T	Non-coding regions	-
149	4741294	C	G	Non-coding regions	-
150	4741296	A	C	Non-coding regions	-
151	4741298	C	T	Non-coding regions	-
152	4754551	C	T	pvdR	ABC transporter permease
153	4760974	C	T	pvdM	dipeptidase
154	4761977	G	A	pvdN	pyoverdine biosynthesis-like

					protein
155	4762818	C	T	pvdO	pyoverdine biosynthesis-like protein
156	4821121	A	G	pvdL	non-ribosomal peptide synthetase
157	4828651	G	A	pvdL	non-ribosomal peptide synthetase
158	4830227	C	T	pvdL	non-ribosomal peptide synthetase
159	4877310	C	T	Non-coding regions	-
160	4886769	G	A	Non-coding regions	-
161	4943847	C	A	Non-coding regions	-
162	4980585	GC	GGGC C	Non-coding regions	-
163	5009829	C	A	PP_4413	hypothetical protein

Figure S1

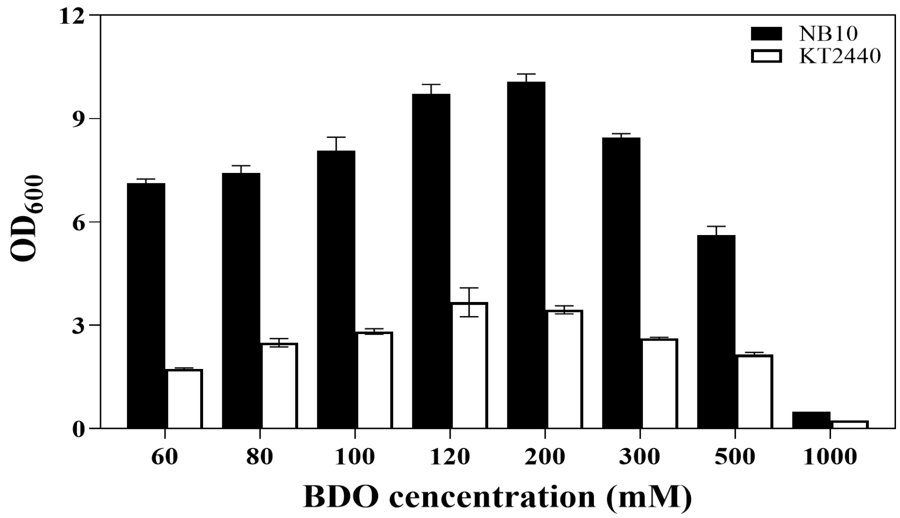

Figure S1. Cell growth of *P. putida* KT2440 and *P. putida* NB10 cultivated with different BDO concentrations as sole carbon source.

Figure S2

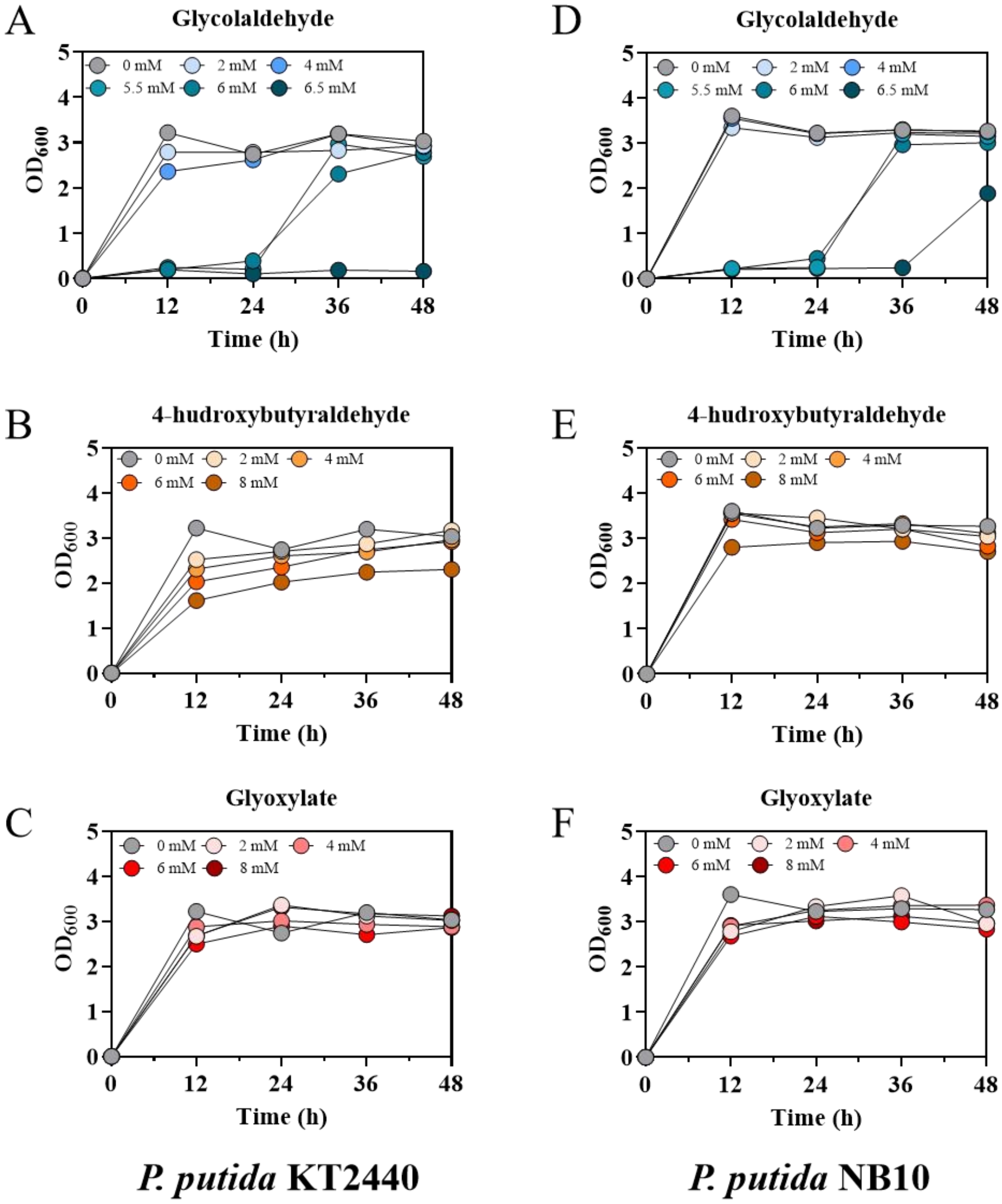

Figure S2. Toxicity test of aldehyde-intermediates towards *P. putida* KT2440 and *P. putida* NB10.

Figure S3

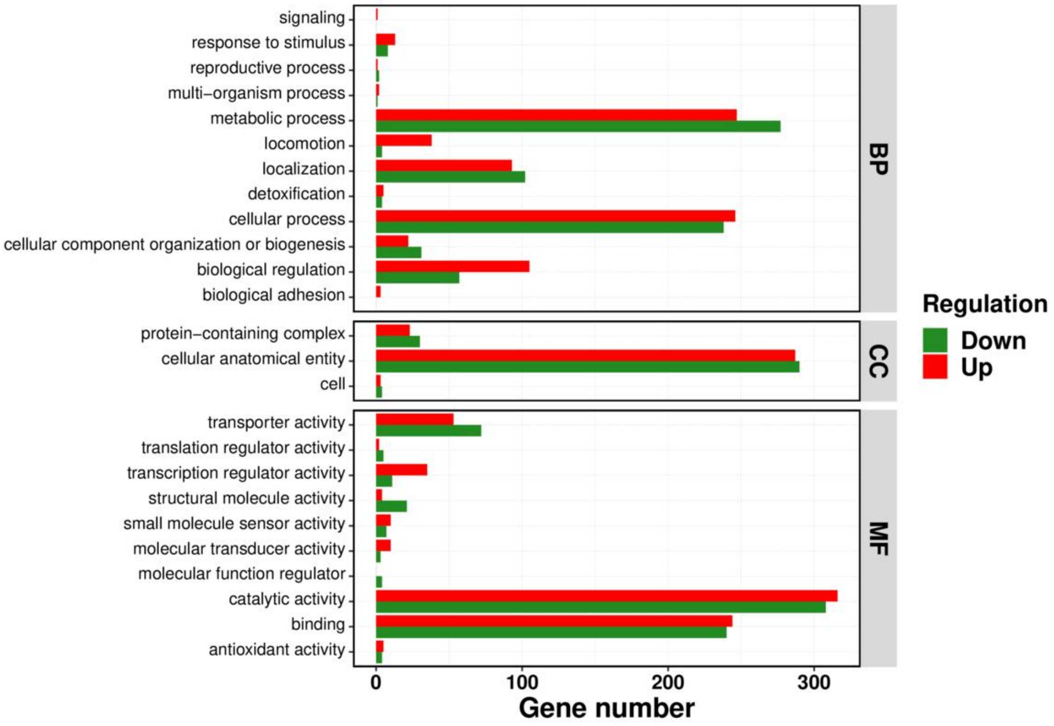

Figure S3. DEGs in GO level 2 of NB10 vs KT2400.

Figure S4

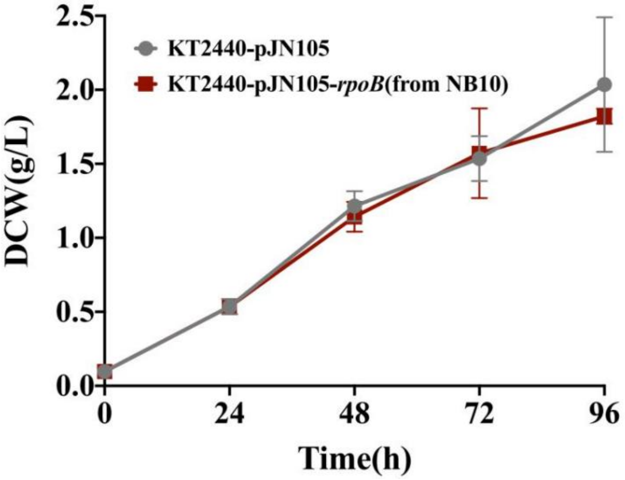

Figure S4. Cell growth of KT2440 expressed the mutant *rpoB* from NB10 with BDO as sole carbon source.

Figure S5

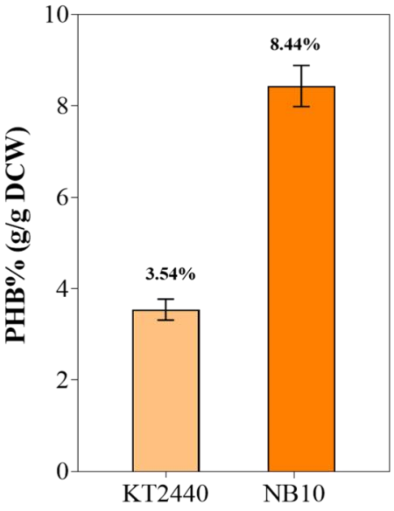

Figure S5. PHB accumulation in *P. putida* NB10 and *P. putida* KT2440.
